# Between-airport heterogeneity in air toxics emissions associated with individual cancer risk thresholds and population risks

**DOI:** 10.1186/1476-069X-8-22

**Published:** 2009-05-08

**Authors:** Ying Zhou, Jonathan I Levy

**Affiliations:** 1 Department of Environmental and Occupational Health, Rollins School of Public Health, Emory University, 1518 Clifton Road NE, Atlanta, Georgia 30322, USA; 2 Department of Environmental Health, Harvard School of Public Health, Landmark Center 4th Floor West, PO Box 15677, Boston, Massachusetts, 02215, USA

## Abstract

**Background:**

Airports represent a complex source type of increasing importance contributing to air toxics risks. Comprehensive atmospheric dispersion models are beyond the scope of many applications, so it would be valuable to rapidly but accurately characterize the risk-relevant exposure implications of emissions at an airport.

**Methods:**

In this study, we apply a high resolution atmospheric dispersion model (AERMOD) to 32 airports across the United States, focusing on benzene, 1,3-butadiene, and benzo [a]pyrene. We estimate the emission rates required at these airports to exceed a 10^-6 ^lifetime cancer risk for the maximally exposed individual (emission thresholds) and estimate the total population risk at these emission rates.

**Results:**

The emission thresholds vary by two orders of magnitude across airports, with variability predicted by proximity of populations to the airport and mixing height (R^2 ^= 0.74–0.75 across pollutants). At these emission thresholds, the population risk within 50 km of the airport varies by two orders of magnitude across airports, driven by substantial heterogeneity in total population exposure per unit emissions that is related to population density and uncorrelated with emission thresholds.

**Conclusion:**

Our findings indicate that site characteristics can be used to accurately predict maximum individual risk and total population risk at a given level of emissions, but that optimizing on one endpoint will be non-optimal for the other.

## Background

For hazardous air pollutants (HAPs), even after implementation of the maximum available control technology (MACT) standards for major stationary sources of air pollution, the residual cancer risks associated with air toxics in the United States (US) generally exceed the 10^-6 ^lifetime risk level often considered as a de minimis cancer risk [1,2]. Therefore increasing attention has been paid to various mobile and area sources and other efforts to control residual risks. While a variety of efforts have been implemented and have contributed to risk reductions [3], some source categories which may contribute to air toxics risks in some settings have not been extensively characterized or formally addressed.

Airports represent a complex source type of increasing importance in many areas. Airports do not meet the definition of a major or area source under Section 112 of the Clean Air Act [4], yet include a combination of sources that contribute to air toxics risks. For example, a study of air toxics risks from O'Hare International Airport in Chicago, Illinois (ORD) [5], estimated that cancer risks associated with the airport exceeded 10^-6 ^for a 1000 square mile area surrounding the airport, with a maximum individual risk (MIR) of 10^-4^. Aircrafts, which contributed 87 percent of these risks, are considered mobile sources but are not subject to the requirements of Section 112 [6].

However, modeling risks from airports or from proposed airport expansions can be complex and somewhat uncertain, given the need for accurate emissions inventories and atmospheric dispersion models that address the intricacies of airport emissions (i.e. aircraft emissions that vary over time and space, including vertically). For this reason, some have concluded that currently available data are inadequate to conduct air toxics risk assessments for airports [6]. For airports, even screening analyses can therefore be time consuming and computationally intensive.

In spite of these data and analytical limitations, there is increasing interest among community groups and other stakeholders in including air toxics risks when considering the marginal contribution of airports or proposed airport expansions to health risks [7]. Given this, it would be desirable to be able to quickly but reasonably estimate the emission rate required for a specific airport to reach a given MIR threshold (which we henceforth define as the de minimis individual risk emission threshold, or DMIRET). In principle, the DMIRET would depend on the proximity of populations to runways and taxiways, meteorological conditions, and the proportion of ground-level versus elevated emissions. It would also depend on the characteristics of the pollutant itself, including its potency, chemical reactivity, and whether it is found in the gas or particle phase.

If the DMIRET could be predicted by these and other covariates for a given toxic air pollutant, the likelihood of MIR thresholds being exceeded could be quickly evaluated. This would allow national regulatory agencies to quickly determine which airports would require greater attention and more extensive modeling efforts to address air toxics. In addition, it would allow interested community groups to quickly ascertain whether an airport or airport expansion would likely contribute to air toxics health risks.

However, focusing exclusively on MIR thresholds in making prioritization decisions could be non-optimal. Although many screening-level cancer risk characterizations are driven initially by an individual risk perspective [3], cost-benefit or related analyses would require population risk estimates, i.e. the sum of individual risks. For example, in the evaluation of residual risks for HAPs, if a source/pollutant combination exceeds the MIR threshold, then the number of people at various risk levels and other considerations are utilized in formulating risk management decisions [3]. It would therefore be important to determine whether population risk measures are correlated with the MIR measures. It is possible that a source would have a lower MIR but a greater total population risk, based on the spatial gradient of concentrations, downwind population density, and other factors.

In this study, we determine for 32 airports distributed across the US the minimum aircraft emission rates of three HAPs with differing potencies and chemical characteristics (benzene, 1,3-butadiene, and benzo [a]pyrene) that would lead to a MIR of 10^-6^. We determine whether significant variability exists in these minimum emission rates and develop models to explain any observed variability based on publicly available covariates. We also calculate the total population risk within 50 km of the airport at these minimum emission rates, and we determine which covariates predict these various measures and whether they are correlated with one another. These analyses allow us to consider the likelihood that an emphasis on avoiding MIR thresholds would be an optimal strategy from a population risk perspective.

## Methods

### Airport sample selection

**Figure 1:**
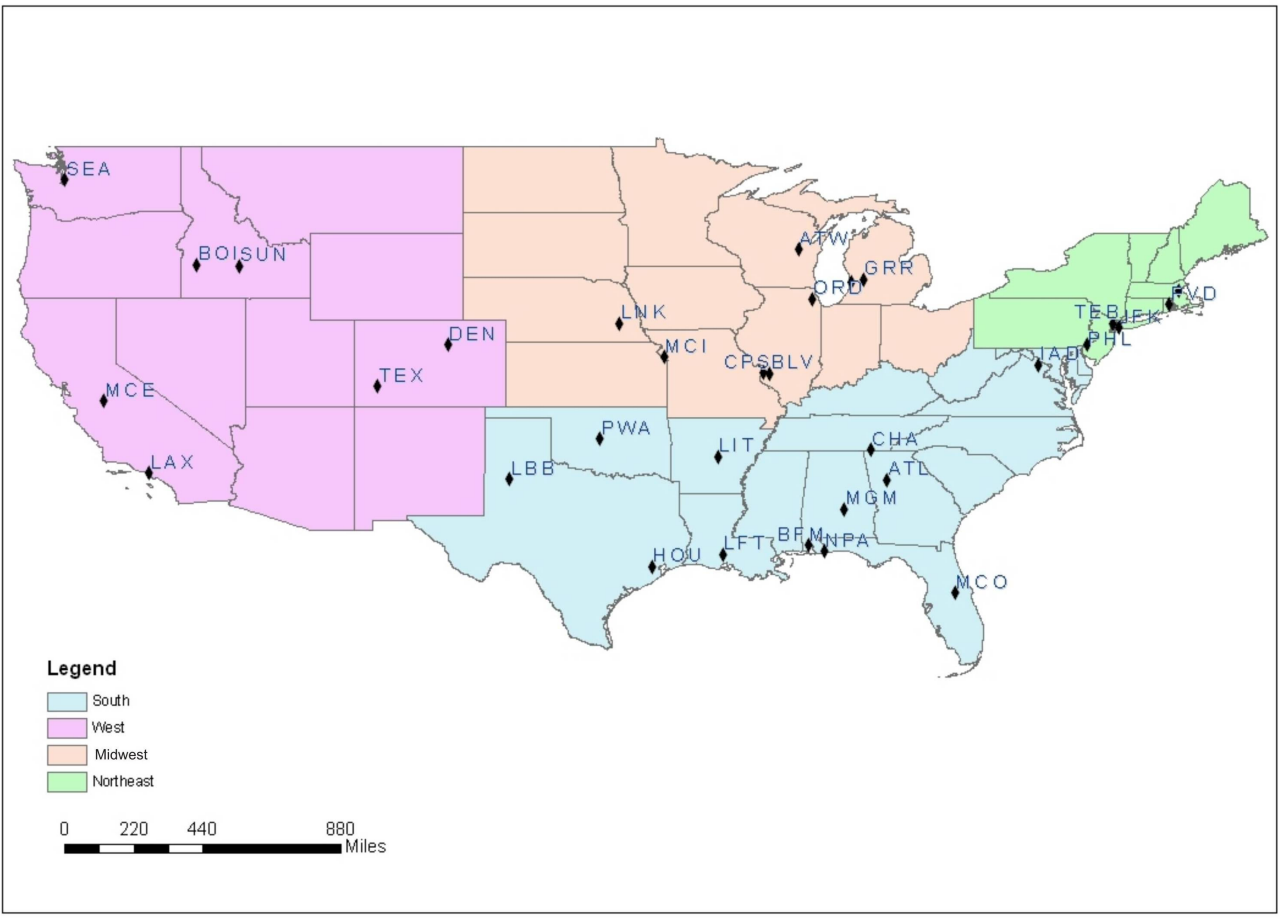
**Location of 32 airports chosen for the analysis**.

As applying detailed atmospheric dispersion models to characterize the marginal effects of all individual airports in the US was infeasible, we instead selected a subset of airports that were representative of the US and adequate to characterize variability in the DMIRET. We began with a set of 325 airports that had been previously characterized using the Emissions and Dispersion Modeling System (EDMS) [8], a combined emissions and dispersion model for assessing air quality at civilian airports and military air bases [9]. These airports represent 95% of commercial jet aircraft operations. We stratified the data set into four census regions – Northeast, Midwest, South and West, as defined by the US Census Bureau [10]. We then randomly selected 10 percent of airports in each region, yielding 5 airports in the Northeast, 8 in the Midwest, 12 in the South, and 7 in the West (Figure 1). Therefore, we obtained a sample of 32 airports for this study, which balanced the need for a large enough sample size for regression analysis with the limitation on computational capacity for air dispersion modeling.

### Atmospheric modeling

We modeled the incremental concentration due to aircraft emissions from airports in the study sample using AERMOD. AERMOD's concentration estimates are based on a steady-state plume approach with significant improvements over previous commonly applied regulatory dispersion models [11,12]. The concentration distribution predicted by AERMOD has been compared with 16 field studies and one laboratory wind tunnel study. With few exceptions, AERMOD's performance is superior to that of the other applied models tested [12]. Breeze AERMOD 6 Graphical User Interface [13] was used to enter input parameters to AERMOD while the executable AERMOD version 07026 by US Environmental Protection Agency (EPA) [14,15] was used to calculate the incremental concentrations.

Several preprocessors are used to generate input data for AERMOD. AERMET is a meteorological data preprocessor that incorporates air dispersion based on planetary boundary layer turbulence structure and scaling concepts. AERMAP is a terrain data preprocessor that incorporates complex terrain using US Geological Survey (USGS) Digital Elevation data. AERSURFACE is a tool that processes land cover data to determine the surface characteristics for use in AERMET.

Surface meteorology and upper air data were obtained from the National Oceanic and Atmospheric Administration (NOAA) [16] for the year 2006. The 1992 National Land Cover Dataset was obtained from USGS from the National Map Seamless Server and was used as input to AERSURFACE. 1 degree terrain elevation data as input to AERMAP were obtained from Trinity Consultants [17].

### Emissions distribution approach

#### Vertical structure

As done previously [9], we modeled the vertical profile of aircraft emissions within seven vertical layers, with data provided by CSSI Inc. The midpoints of these seven layers are at 3, 58, 121, 232, 390, 591 and 837 m. Emissions from engine startup, Auxiliary Power Units (APUs) and aircraft taxi in and out are in layer 1. Aircraft takeoff with initial climb, the climbout and the approach mode are divided among layers 1 to 7. For total hydrocarbon (THC) and volatile organic carbon (VOC) emissions, layer 1 contributes the majority of emissions. For the 32 chosen airports, 87 to 97 percent of THC and VOC emissions are from layer 1 with an average of 94 percent. As a comparison, for carbon monoxide and particulate matter, an average of 74 and 50 percent respectively are from layer 1. Because the contribution by layer did not vary substantially across airports, for the air toxics under study in this analysis, we modeled the contribution of each layer following the average percentage contribution for THC and VOC. This corresponded to 93.7%, 0.3%, 0.5%, 1.1%, 1.1%, 1.6%, and 1.6% from the first layer to the seventh layer respectively.

We treat the emissions within each layer as an area source, with the first layer modeled as a polygon area source approximating the shape of the airport and the rest of the layers modeled as circular area sources. The radius of the top layer is assumed to be 20 km – the horizontal threshold in EDMS. We approximate the radius of the bottom layer as 5 km, although it is modeled as a polygon area source with variable configurations across airports, and the radii of the layers in between were calculated by projecting the bottom layer to the top layer and assuming a cone-like shape. In the end, the top two layers are combined into one due to limitations within AERMOD for modeling area sources above 700 m. Therefore, we have a total of six layers with the highest layer at 591 m with a radius of 15.6 km.

#### Temporal emission distribution

Modeling detailed hourly emissions for each individual airport was infeasible, so we used the approach from EDMS [18], which modeled the temporal emission profiles of three airports – Providence/T.F. Green International Airport in Warwick, RI (PVD), Hartsfield-Jackson Atlanta International Airport (ATL), and Chicago O'Hare International Airport (ORD) – and developed rules for mapping other airports to these three airports [19]. For example, if the airport has less than or equal to the number of commercial operations that PVD has, then it was mapped to PVD, with the same relative emissions patterns. If the airport has more crossing runways than parallel runways, it was mapped to ORD. Otherwise, it was mapped to ATL. For the airports in the study sample, there are 7 with ATL type, 5 with ORD type and 20 with PVD type emission profiles (See Figure S1 in Additional File 1).

#### Receptor selection

We modeled pollutant dispersion within 50 km of each airport of interest, using the discrete receptor setting in AERMOD. This radius would be expected to capture the MIR, as the MIR would likely occur near the airport, but would not go beyond the recommended modeling distance for AERMOD. Although not all total population exposure would occur within this radius, a significant enough portion would generally be found to evaluate our core hypotheses. Within 5 km of the airport, a higher receptor density is used with receptor locations being the centroids of census block groups. Between 5 and 50 km of the airport, the receptors are the centroids of census tracts. Population data are based on year 2000 US Census data [20]. For the airports in the study sample, the number of receptors within 50 km of the airport ranges from less than 20 (for SUN in Idaho and TEX in Colorado) to nearly 4,000 (for JFK in New York and TEB in New Jersey) with an average of about 700.

#### Pollutants modeled

We focus on three air toxics with different chemical characteristics – benzene, 1,3-butadiene, and benzo [a]pyrene (BaP). We use benzene to represent conservative air toxics (i.e. non-reactive), 1,3-butadiene to represent reactive air toxics, and BaP to represent particulate air toxics (as a particle-bound polycyclic aromatic hydrocarbon). For 1,3-butadiene, modeling complex chemical reactions is beyond the scope of AERMOD. Instead, we assumed a half life of 2 hours, its half life reported in sunlight [21], to determine whether this leads to qualitatively different conclusions than seen for conservative air toxics. Both dry and wet deposition of BaP are modeled. For dry deposition, a mass median diameter of 0.1 μm with a fine mass fraction of 0.93 is used, based on the recommended values for polycyclic organic compounds from Appendix B of the report on Deposition Parameterizations for the Industrial Source Complex (ISC3) Model [22].

#### Cancer potency factors

For the three selected pollutants, we relied on standard inhalation unit risks to estimate health risks. Benzene is a known human carcinogen which has been associated with leukemia and other neoplastic conditions. Within the EPA's Integrated Risk Information System (IRIS) database, the inhalation unit risk of benzene was reported as a range, with values between 2.2 × 10^-6 ^and 7.8 × 10^-6 ^for lifetime exposure to 1 μg/m^3 ^benzene in air [23]. Given the nature of our analysis, for which the core variability calculations and models are not dependent on the chosen cancer potency factor (as risks scale linearly with potency), we selected the average of this range of values (5 × 10^-6^) for our potency estimate and do not formally address uncertainties within our primary analyses.

1,3-butadiene is also considered by the EPA to be a known human carcinogen, with an inhalation unit risk based on epidemiological evidence. The most recent value reported in IRIS is 3 × 10^-5 ^for lifetime exposure to 1 μg/m^3 ^in air [23]. Finally, BaP does not have an inhalation unit risk in the IRIS database, so we relied on an assessment conducted by the California Office of Environmental Health Hazard Assessment (OEHHA). OEHHA considered BaP to be genotoxic, and developed an inhalation unit risk of 1.1 × 10^-3 ^for lifetime exposure to 1 μg/m^3 ^in air based on a study of respiratory tract tumors in hamsters [24]. We recognize that BaP's risks may be influenced significantly by non-inhalation pathways, but focus herein on inhalation, given that BaP is being used as a representative of particle-bound compounds rather than because of specific interest in BaP.

#### Analytical framework

For each of the pollutants and airports, we estimate the DMIRET, the total population risk at that level of emissions, and the population intake fraction (defined below). To estimate the DMIRET, we first identify the receptor location (i.e. census block group) in the modeling domain with the highest incremental concentration from aircraft emissions. This concentration is then combined with the corresponding cancer potency factor to estimate the increase in maximum individual cancer risk. Since AERMOD does not include non-linear atmospheric chemistry, we can then back-calculate the emission rate corresponding to the maximum individual risk threshold of 10^-6^, which is defined as the DMIRET.

We can then adjust the incremental concentration outputs at all receptors within 50 km of the airport to correspond with the DMIRET, and can directly estimate population cancer risk as the sum across receptors of population multiplied by incremental concentration, multiplied by the cancer potency factor. A component of this calculation is the total population exposure within 50 km of the airport per unit emissions, which we summarize using the metric of intake fraction (iF) – the fraction of a material released from a source that is inhaled or ingested [25]. We calculate iF by combining marginal concentration (C_i_) and population count (P_i_) at corresponding receptors within 50 km from the airport times a nominal breathing rate (BR) of 20 m^3 ^per day divided by emission rate (Q), which can be represented as iF = (Σ C_i _× P_i_)× BR/Q. As none of the three pollutants studied have meaningful in-situ formation, this will capture population exposure per unit emissions for these pollutants. Once iF has been calculated, population risks at the DMIRET can be easily obtained by combining the emission rate (i.e. the DMIRET), iF, cancer potency factor, and nominal breathing rate. In our regression analyses, we consider predictors of variability in iF as well as the DMIRET, so that both individual risk and population risk findings from this study can be extrapolated to other airports not included in this study sample.

#### Regression analysis independent variables

To help explain variability in the DMIRET and iF, we summarized several independent variables to represent local meteorology, population near the airport, and distance from the airport to the nearest receptor. Meteorological variables include mixing height and wind speed. Three different ways of incorporating mixing heights are tested – annual average mixing height, the annual average of the maximum daily mixing height, and the harmonic mean mixing height (which theoretically captures the inverse relationship between mixing height and concentrations). For the population variable in the iF regression, we use total population within 50 km of the airport. For the DMIRET regression, we consider two different ways of calculating the distance between the airport and the nearest receptor: the distance from the airport centroid to the nearest receptor, and the distance from the airport fenceline to the nearest receptor. We note that the nearest receptor may not be the receptor with the maximum individual risk, but this represents a variable available for an airport prior to conducting any dispersion modeling. Table S1 (see Additional file 2) summarizes the values of these independent variables.

## Results and discussion

### Summary statistics

Table 1 lists the emission thresholds (DMIRET) corresponding to 1 × 10^-6 ^cancer risks for benzene, 1,3-butadiene and BaP at the maximally exposed receptor location across the 32 airports as well as the summary statistics such as the mean, standard deviation, minimum and maximum. Intake fractions are also listed in Table 1 for comparison. First considering the DMIRET, there is approximately 100-fold variation across airports for all three pollutants. The mean DMIRET is 10, 2 and 0.05 metric tons per year for benzene, 1,3-butadiene and BaP respectively, but values at individual airports differ from the mean by an order of magnitude in either direction. Of note, the maximum individual risk occurs at the same receptor for all three pollutants at all airports. This receptor is the receptor with the minimum distance to the airport in many cases, or one of the receptors with the closest distances in the rest of the cases.

**Table 1:** Intake fraction (iF) and de minimis individual risk emission threshold (DMIRET) values for the 32 airports, reported to two significant figures.

	iF			DMIRET		
		
Airport	Benzene	1,3-butadiene	BaP	Benzene (metric ton/year)	1,3-butadiene (metric ton/year)	BaP (kg/year)
	
ATL	8.2E-06	4.3E-06	7.8E-06	5.0	0.86	23
	
ATW	1.6E-06	9.3E-07	1.6E-06	39	7.7	180
	
BFM	7.1E-06	5.0E-06	6.8E-06	0.82	0.14	3.8
	
BIV	2.3E-06	1.6E-06	2.2E-06	6.3	1.1	29
	
BLV	2.0E-06	7.2E-07	1.8E-06	21	6.0	100
	
BOI	1.1E-05	7.0E-06	1.0E-05	2.7	0.46	12
	
BOS	1.4E-05	1.0E-05	1.4E-05	3.0	0.51	14
	
CHA	4.7E-06	3.6E-06	4.5E-06	1.6	0.27	7.3
	
CPS	1.1E-05	7.0E-06	1.0E-05	1.4	0.24	6.3
	
DEN	2.1E-06	8.4E-07	1.9E-06	14	2.6	66
	
GRR	2.5E-06	1.3E-06	2.4E-06	24	4.8	110
	
HOU	1.9E-05	1.1E-05	1.8E-05	1.9	0.32	8.6
	
IAD	9.9E-06	4.4E-06	9.1E-06	10	1.9	47
	
JFK	5.8E-05	3.3E-05	5.5E-05	4.0	0.71	19
	
LAX	3.4E-05	2.0E-05	3.2E-05	3.9	0.69	18
	
LBB	4.8E-07	3.0E-07	4.6E-07	17	2.9	76
	
LFT	5.6E-06	3.5E-06	5.3E-06	5.8	1.1	27
	
LIT	3.5E-06	2.6E-06	3.4E-06	2.8	0.47	13
	
LNK	3.2E-06	2.3E-06	3.0E-06	3.1	0.53	14
	
MCE	4.6E-06	3.6E-06	4.4E-06	2.3	0.40	11
	
MCI	2.4E-06	1.1E-06	2.2E-06	7.3	1.3	34
	
MCO	7.4E-06	2.8E-06	6.9E-06	9.6	1.8	45
	
MGM	3.6E-06	1.8E-06	3.3E-06	4.5	0.88	21
	
NPA	5.0E-06	3.0E-06	4.7E-06	1.8	0.31	8.2
	
ORD	2.6E-05	1.4E-05	2.4E-05	5.6	0.99	26
	
PHL	1.9E-05	1.1E-05	1.8E-05	3.4	0.58	16
	
PVD	1.2E-05	8.3E-06	1.2E-05	1.4	0.24	6.6
	
PWA	4.8E-06	3.5E-06	4.6E-06	3.0	0.51	14
	
SEA	1.2E-05	7.1E-06	1.2E-05	1.8	0.31	8.4
	
SUN	1.7E-07	1.3E-07	1.7E-07	14	2.5	65
	
TEB	2.9E-05	1.6E-05	2.7E-05	1.9	0.32	8.6
	
TEX	5.2E-08	2.7E-08	4.9E-08	110	30	520
	
**Mean**	1.0E-05	6.0E-06	9.6E-06	10	2.3	49
	
**Min**	5.2E-08	2.7E-08	4.9E-08	0.82	0.14	3.8
	
**Max**	5.8E-05	3.3E-05	5.5E-05	110	30	520
	
**SD**	1.2E-05	6.9E-06	1.2E-05	20	5.4	94

The mean intake fractions for the three pollutants modeled are on the order of 10^-5^, meaning that for every metric ton of aircraft pollutants emitted from airports, on average 10 g is inhaled by all residents within 50 km of the airport. Although the 50 km radius somewhat complicates comparisons with studies generally using larger radii, these values are on average slightly greater than previously reported for primary pollutants from power plants [26,27] and similar to those previously reported for mobile sources [28,29]. This would be anticipated given that 94% of VOC emissions from airplanes are at ground level, similar to mobile sources, while power plants usually have tall stack heights. For iF, the variation across airports is even larger than for DMIRET, with an approximate 1000-fold difference between the minimum and the maximum. It should be noted that a high iF indicates that a unit change in emissions would have a greater influence on total population risk, given the greater total population exposure, while a low DMIRET indicates that a unit change in emissions would have a greater influence on maximum individual risk.

Among the three pollutants studied, benzene has the highest iF, as it is modeled as a conservative pollutant. BaP is generally similar to benzene, with somewhat lower values for 1,3-butadiene, indicating that the removal rate due to wet and dry deposition for BaP is somewhat less than due to chemical reactions for 1,3-butadiene. Considering the pollutant concentrations at the same emission rates, the average ratio of 1,3-butadiene to benzene across all the different receptor locations in the modeling domain is 0.43, versus 0.92 for the average ratio of BaP to benzene. As expected, the ratios for the receptors closer to the airport are close to 1 (ratios of 0.90 and 0.97 for 1,3-butadiene to benzene and BaP to benzene, respectively), while the same ratios for receptors about 50 km from the airport are 0.22 and 0.87, respectively. This emphasizes that pollutant characteristics will have a smaller effect on maximum individual risk than on population risk.

We can estimate the population cancer risk at the DMIRET for each airport, which addresses the question of whether having the identical maximum individual cancer risk across airports would lead to similar population risks. The population cancer risk at the DMIRET can be calculated as DMIRET × iF × potency factor/BR. The population cancer risk at the DMIRET varies by nearly two orders of magnitude across airports (factor of 99 difference between minimum and maximum population risk for benzene, factor of 71 difference for 1,3-butadiene, and factor of 93 difference for BaP). The airports with the highest population risk at the DMIRET are those that have a high iF, such as JFK, ORD, and LAX, and the population risk is not significantly correlated with the DMIRET itself (correlation coefficient of -0.09 for benzene, p = 0.62).

Another way of considering the difference in prioritization between a population risk and maximum individual risk approach is to consider the implications of a unit change in emissions on both endpoints. For example, at JFK, a one metric ton/year increase in benzene emissions would result in a population risk increase of 0.04 lifetime cancer cases (the highest value across all airports), as the product of an iF of 5.8 × 10^-5 ^and the potency of 5 × 10^-6 ^per μg/m^3^, divided by the nominal breathing rate of 20 m^3^/day with appropriate unit conversions. As a comparison, the one metric ton/year increase in benzene emissions would result in a maximum individual risk increase of 2.5 × 10^-7^, given a DMIRET of 4 metric tons/year (corresponding to a maximum individual risk of 10^-6^). This is near the median of the maximum individual risk increase across airports. Figure S2 (see Additional file 3) demonstrates the generally weak association between the population risk increase and maximum individual risk increase per unit increase in benzene emissions. This is driven by the relatively weak correlation between the iF and the DMIRET (the correlation coefficient between these two measures for benzene is -0.27, p = 0.13). This is not surprising as different factors influence total population exposure and maximum individual exposure, which we analyze more systematically in the regression analysis.

### Regression analysis

In univariate regressions, the most significant predictor of DMIRET is distance to airport, with greater significance for distance from airport centroid to receptors. There is a non-linear relationship between DMIRET and distance, which is anticipated given standard Gaussian dispersion concepts, in which the relationship between pollutant concentration and downwind distance is reflected in the dispersion coefficient(s). We tried different transformations on the distance variable as well as on the DMIRET (dependent variable), of which the log transformation on the DMIRET turns out to work best. Figure S3 (see Additional file 4) shows how the log-transformed benzene DMIRET increases approximately linearly with distance. The plots for 1,3-butadiene and BaP are similar to that for benzene. In multivariate models (Table 2), both the distance variable and a log-transformed annual average mixing height variable are significant (p < 0.05). These regressions explain 74–75% of the variability in DMIRET across the three pollutants. The log transformation on mixing height improves the model fit, and there is no significant difference in model fit using the three different mixing height measures.

**Table 2:** Parameter estimates for de minimis individual risk emission threshold regressions for different pollutants.

Dependent Variable	Independent Variables			
			
	Distance between nearest census block group centroid and airport centroid (km)	Log annual average mixing height (m)	Intercept	**R**^**2**^
Log benzene emission threshold (kg/year)	0.683 (<0.0001)	1.43 (0.019)	-1.65 (0.66)	0.74

Log 1,3-butadiene emission threshold (kg/year)	0.751 (<0.0001)	1.40 (0.028)	-3.23 (0.41)	0.75

Log BaP emission threshold (g/year)	0.687 (<0.0001)	1.42 (0.021)	-0.019 (0.995)	0.74

P-values are listed below regression coefficients.

Turning to intake fractions, given the definition of iF, we construct no-intercept models considering total population and the product of population and mixing height (since intake fraction should be zero if there is no exposed population). As anticipated, total population is a highly significant predictor, and the product of population and average daily mixing height is also significant at the p < 0.05 level (Table 3). The final regression equations therefore reinforce that iF will increase linearly with population, but with a slope that is lower in areas with greater mixing heights and therefore lower concentrations per unit emissions. These regressions explain 93–95% of the variability in intake fraction across the three pollutants, although the R^2 ^should be interpreted with care for no-intercept models.

**Table 3:** Parameter estimates for intake fraction regressions for different pollutants.

Dependent Variable	Independent Variables		R^2^
		
	Population within 50 km of the airport	Product of Population and annual average mixing height (m)	
Benzene iF	8.18 × 10^-12^ (<0.0001)	-7.05 × 10^-15^ (<0.0001)	0.95

1,3-butadiene iF	4.78 × 10^-12^ (<0.0001)	-4.19 × 10^-15^ (<0.0001)	0.93

BaP iF	7.69 × 10^-12^ (<0.0001)	-6.59 × 10^-15^ (<0.0001)	0.95

P-values are listed below regression coefficients.

Figure S4 (See Additional file 5) shows how benzene intake fraction increases approximately linearly with population within 50 km from airports. For 1,3-butadiene and BaP, the plots are similar, but with slightly different slopes. The one outlier from this linear relationship is TEB (Teterboro, New Jersey). This can be explained by the fact that TEB is in a relatively less populated area but is within 50 km of New York City. Thus, it has a similar total population within 50 km as JFK (the other high-population point on Figure S4), but that population is disproportionately found at longer distances from the airport where incremental concentrations from TEB are lower. If we had constructed regressions including population within various radii, our predictive power would have increased further, but we retain the model shown in Table 3 to be parsimonious.

### Uncertainty and sensitivity analyses

Although the DMIRET and iF values in Table 1 are presented without uncertainty bounds, numerous factors contribute uncertainty to these values. Meteorological factors, airport emissions characterization, and other atmospheric dispersion model inputs influence both values, and the DMIRET is also affected by the assumed cancer potency value. As the DMIRET will scale linearly with potency, uncertainty bounds could be readily calculated if the uncertainties in potency were fully characterized. This could allow a decision maker to determine, for example, the emission rate that would not exceed a 10^-6 ^maximum individual cancer risk with 95% confidence. In addition, in situations where potency ranges are reported (as for benzene), it could be determined whether the emissions from an airport would exceed a 10^-6 ^maximum individual cancer risk for any of the values within that range. As the range for benzene does not reflect a formal confidence interval and no such uncertainty characterization is available for the other air toxics, we do not formally incorporate uncertainty in potency, but recognize that the DMIRET values in Table 1 should be interpreted with caution given these uncertainties.

In addition, to illustrate some of the uncertainties associated with our atmospheric modeling, we conducted sensitivity analyses for concentration estimates from one airport (PVD) using meteorological input from a different year and characterizing airport emissions as a volume source rather than as an area source. Note that a volume source is essentially an area source with a third dimension of height. When meteorological data for 2007 are used, the maximum annual average concentration found in the modeling domain is 44 percent lower than the base case (meteorological data from 2006), possibly due to a combination of faster wind speed but lower mixing height that were observed in year 2007. This means that the MIR is 44 percent lower and the corresponding DMIRET will be 79 percent (1/0.56) higher. The population intake fraction is 46 percent lower than the base case, possibly mainly due to the lower mixing height that was observed in year 2007 and which translates to 46 percent lower population risk for the same emission rate. When a volume source is used instead of an area source, the results are most sensitive to settings in the first layer, where more than 90 percent of the emissions are from. For example, the size of the lateral dimension of the volume source in the first layer can change the maximum concentration in the domain by as much as 68 percent from the base case value. The corresponding intake fraction values are not as sensitive to the volume source parameter settings, which stayed within 10 percent of the base case value. While these quantitative results do not necessarily generalize to all airports, they emphasize that the DMIRET and population risk estimates should not be considered as absolute values, but would vary across years and include uncertainties beyond the potency uncertainties described above.

### Limitations

Multiple limitations influence the interpretation of our findings. First, our analyses only characterized the emission rates from aircraft that would lead to 10^-6 ^maximum individual risk for individual air toxics, omitting non-aircraft sources at the ground level and the cumulative effect of multiple exposures. However, previous studies [5] have shown that aircraft dominate the air toxics risks from airports, the vertical emissions profile indicates that model outputs for ground-level sources would be similar, and our methods are readily generalizable to a cumulative risk framework. The fact that the DMIRET was highly correlated between pollutants with differing chemical characteristics (correlation coefficient > 0.99 for all three pollutants we studied) indicates that model outputs for one pollutant could be readily extrapolated to other pollutants without complex chemical reactions or extensive in-situ formation. Similarly, given the linearity in the system, the emission threshold associated with other individual risk levels of interest could be quickly ascertained. Treating all aircraft emissions as area sources clearly omits some important spatial heterogeneity, especially given the runway configurations and correlation between flight patterns and wind direction, and modeling the time-varying emissions of individual aircraft at all airports was well beyond the scope of this study. Our methodology is clearly generalizable, but the magnitude and location of the MIR could differ if these complexities were taken into account.

In addition, as we were lacking comprehensive emission inventories for all airports, we could not directly interpret the DMIRET in relation to the actual or anticipated emission rates, which complicates interpretability. In other words, although the DMIRET is lowest for BaP given its potency, the emissions of BaP would be anticipated to be much lower than the emissions of benzene or 1,3-butadiene. Preliminary examination of estimated air toxics emissions for PVD, ORD, and ATL suggest that current emissions from these airports would exceed a 10^-6 ^MIR for benzene and 1,3-butadiene but not BaP, but more comprehensive analyses (including formal examination of key sensitivities and uncertainties) would be needed to draw policy-relevant conclusions for these and other airports. If flight activity proved to be a reasonable proxy of emissions, this could provide another indicator that could be combined with estimates of DMIRET and iF to yield rapid yet reasonable interpretations. More generally, useful conclusions could potentially be drawn even for airports lacking comprehensive emissions inventories. For example, if a very small airport would need to have emissions greater than those from a very large airport to exceed a defined MIR threshold, it could be concluded that the MIR threshold would not likely be exceeded.

## Conclusion

In spite of these limitations, our analyses corroborate our hypotheses and demonstrate the viability of our approach. Using state-of-the-art four-dimensional emissions characterization and atmospheric dispersion modeling, we demonstrated that both the emission rate contributing to a 10^-6 ^maximum individual risk and the total population exposure within 50 km of the airport per unit emissions vary substantially across airports but can be predicted with reasonable precision using easy to obtain variables, such as distance from the airport, total population, and mixing height. These results provide a method to quickly but reasonably determine the likelihood of public health impacts of concern for airport modifications or expansions. In addition, there is low correlation between the emission rate contributing to a 10^-6 ^maximum individual risk and the total population risk within 50 km of the airport at that emission rate, emphasizing that decisions based solely on one factor may not be optimal for the other factor. Our methods can be generalized to other source categories and can be expanded to include other pollutants with non-threshold dose-response curves in future assessments.

## Abbreviations

ATL: Hartsfield-Jackson Atlanta International Airport; BaP: Benzo [a]pyrene; BR: Breathing rate; DMIRET: de minimis individual risk emission threshold; EDMS: Emissions and Dispersion Modeling System; EPA: Environmental Protection Agency; HAP: Hazardous air pollutants; iF: Intake Fraction; IRIS: Integrated Risk Information System; ISC: Industrial Source Complex model; JFK: John F. Kennedy International Airport in New York City; LAX: Los Angeles International Airport in Los Angeles; MACT: Maximum available control technology; MIR: Maximum individual risk; NOAA: National Oceanic and Atmospheric Administration; OEHHA: Office of Environmental Health Hazard Assessment; ORD: O'Hare International Airport in Chicago; PVD: Providence/T.F. Green International Airport in Warwick, RI; SUN: Friedman Memorial Airport in Hailey, Idaho; TEB: Teterboro Airport in Teterboro, New Jersey; TEX: Telluride Regional Airport in Telluride, Colorado; THC: Total hydrocarbon; USGS -US Geological Survey; VOC: Volatile organic carbon

## Competing interests

The authors declare that they have no competing interests.

## Authors' contributions

YZ collected input data and carried out the atmospheric modeling, conducted the statistical analysis and drafted the manuscript. JIL conceived of the study, helped refine the analysis and revised the manuscript. All authors read and approved the final manuscript.

## Supplementary Material

Additional file 1 **Relative emission profiles by hour of day and day of week for three template airports**. The plots in this file show the relative emission profile by hour of day as well as day of week for ATL, ORD and PVD. Note that all emissions are relative to the maximum value by hour of day or day of week, which is assigned a value of 1.0. Click here for file

Additional file 2 **Distribution of independent variables and summary statistics at 32 airports**. The data in this table summarize the values of the independent variables at the 32 airports under study. Click here for file

Additional file 3 **Increase in maximum individual cancer risk and total population cancer risk within 50 km of the airport from benzene for a 1 metric ton/year increase in emissions at each airport**. This figure demonstrates the generally weak association between the population risk increase and maximum individual risk increase per unit increase in benzene emissions. Click here for file

Additional file 4 **Benzene de minimis individual risk emission threshold with a natural logarithm transformation vs. distance between the nearest census block centroid and the airport centroid**. This figure shows the log-transformed benzene DMIRET increases approximately linearly with distance. Click here for file

Additional file 5 **Benzene intake fractions vs. population within 50 km of airports**. This figure shows how benzene intake fraction increases approximately linearly with population within 50 km from airports. Click here for file
