# Supplementary material for: Between-airport heterogeneity in air toxics emissions associated with individual cancer risk thresholds and population risks
Source: Environ Health. 2009 May 8;8:22. doi: 10.1186/1476-069X-8-22 (PMC2687437; doi:10.1186/1476-069X-8-22)
Supplement: Additional file 1 — Relative emission profiles by hour of day and day of week for three template airports. The plots in this file show the relative emission profile by hour of day as well as day of week for ATL, ORD and PVD. Note that all emissions are relative to the maximum value by hour of day or day of week, which is assigned a value of 1.0. [file 1476-069X-8-22-S1.doc]

Figure S1 Relative emission profiles by hour of day and day of week for three template airports. All emissions are relative to the maximum value by hour of day or day of week, which is assigned a value of 1.0.
