# Supplementary material for: Between-airport heterogeneity in air toxics emissions associated with individual cancer risk thresholds and population risks
Source: Environ Health. 2009 May 8;8:22. doi: 10.1186/1476-069X-8-22 (PMC2687437; doi:10.1186/1476-069X-8-22)
Supplement: Additional file 2 — Distribution of independent variables and summary statistics at 32 airports. The data in this table summarize the values of the independent variables at the 32 airports under study. [file 1476-069X-8-22-S2.doc]

Table S1 Distribution of independent variables and summary statistics at 32 airports

| Airport | Distance between nearest census block group centroid and airport boundary (m) | Distance between nearest census block group centroid and airport centroid (m) | Annual average wind speed (m/s) | Annual average mixing height (m) | Annual average max daily mixing height (m) | Harmonic mean mixing height (m) | Population within 50 km of the airport |
| --- | --- | --- | --- | --- | --- | --- | --- |
| ATL | 55 | 362 | 4.70 | 689 | 1,566 | 374 | 3,416,317 |
| ATW | 1,898 | 3,029 | 3.95 | 572 | 1,186 | 179 | 538,252 |
| BFM | 371 | 720 | 3.19 | 371 | 820 | 119 | 569,499 |
| BIV | 610 | 1,014 | 4.24 | 682 | 1,369 | 239 | 583,568 |
| BLV | 1,889 | 2,736 | 3.05 | 683 | 1,512 | 126 | 1,047,687 |
| BOI | 429 | 409 | 3.57 | 800 | 1,894 | 228 | 465,547 |
| BOS | 99 | 226 | 4.93 | 629 | 1,249 | 269 | 3,922,403 |
| CHA | 224 | 1,105 | 2.30 | 662 | 1,361 | 133 | 750,972 |
| CPS | 46 | 606 | 3.28 | 581 | 1,244 | 156 | 2,244,068 |
| DEN | 2,666 | 1,293 | 4.70 | 854 | 1,927 | 388 | 2,243,995 |
| GRR | 619 | 2,550 | 4.22 | 644 | 1,322 | 234 | 916,360 |
| HOU | 299 | 79 | 3.29 | 617 | 1,316 | 166 | 3,960,952 |
| IAD | 37 | 3,529 | 3.00 | 595 | 1,266 | 151 | 3,590,843 |
| JFK | 40 | 82 | 3.00 | 641 | 1,324 | 181 | 13,730,382 |
| LAX | 5 | 306 | 3.31 | 581 | 1,307 | 189 | 9,440,506 |
| LBB | 35 | 2,072 | 5.37 | 819 | 1,765 | 324 | 295,054 |
| LFT | 666 | 1,831 | 2.85 | 602 | 1,249 | 157 | 520,733 |
| LIT | 660 | 131 | 3.36 | 541 | 1,213 | 150 | 631,580 |
| LNK | 77 | 705 | 4.47 | 617 | 1,331 | 175 | 327,984 |
| MCE | 49 | 681 | 2.47 | 496 | 1,042 | 169 | 376,271 |
| MCI | 746 | 1,330 | 4.67 | 625 | 1,288 | 249 | 1,612,914 |
| MCO | 438 | 2,590 | 3.43 | 522 | 1,179 | 173 | 1,718,472 |
| MGM | 472 | 1,427 | 2.64 | 512 | 1,214 | 117 | 390,341 |
| NPA | 455 | 356 | 3.44 | 488 | 940 | 169 | 476,226 |
| ORD | 10 | 1,047 | 4.13 | 575 | 1,198 | 184 | 7,553,560 |
| PHL | 23 | 1,454 | 4.21 | 620 | 1,279 | 241 | 5,098,734 |
| PVD | 94 | 18 | 3.98 | 562 | 1,227 | 171 | 1,786,300 |
| PWA | 80 | 775 | 5.32 | 858 | 1,613 | 308 | 1,030,918 |
| SEA | 27 | 243 | 3.65 | 549 | 1,137 | 232 | 2,980,562 |
| SUN | 503 | 1,598 | 3.04 | 654 | 1,363 | 264 | 35,212 |
| TEB | 12 | 710 | 3.31 | 858 | 1,701 | 271 | 13,955,867 |
| TEX | 5,639 | 6,563 | 2.36 | 594 | 1,505 | 176 | 58,216 |
| Mean | 602 | 1,299 | 3.7 | 628 | 1,341 | 208 | 2,714,139 |
| Min | 5 | 18 | 2.3 | 371 | 820 | 117 | 35,212 |
| Max | 5,639 | 6,563 | 5.4 | 858 | 1,927 | 388 | 13,955,867 |
| SD | 1,108 | 1,346 | 0.8 | 113 | 245 | 70 | 3,647,146 |
