# Supplementary material for: Between-airport heterogeneity in air toxics emissions associated with individual cancer risk thresholds and population risks
Source: Environ Health. 2009 May 8;8:22. doi: 10.1186/1476-069X-8-22 (PMC2687437; doi:10.1186/1476-069X-8-22)
Supplement: Additional file 3 — Increase in maximum individual cancer risk and total population cancer risk within 50 km of the airport from benzene for a 1 metric ton/year increase in emissions at each airport. This figure demonstrates the generally weak association between the population risk increase and maximum individual risk increase per unit increase in benzene emissions. [file 1476-069X-8-22-S3.doc]

Figure S2 Increase in maximum individual cancer risk and total population cancer risk within 50 km of the airport from benzene for a 1 metric ton/year increase in emissions at each airport.
