# Supplementary material for: Between-airport heterogeneity in air toxics emissions associated with individual cancer risk thresholds and population risks
Source: Environ Health. 2009 May 8;8:22. doi: 10.1186/1476-069X-8-22 (PMC2687437; doi:10.1186/1476-069X-8-22)
Supplement: Additional file 4 — Benzene de minimis individual risk emission threshold with a natural logarithm transformation vs. distance between the nearest census block centroid and the airport centroid. This figure shows the log-transformed benzene DMIRET increases approximately linearly with distance. [file 1476-069X-8-22-S4.doc]

Figure S3 Benzene de minimis individual risk emission threshold with a natural logarithm transformation vs. distance between the nearest census block centroid and the airport centroid
