# Supplementary material for: Between-airport heterogeneity in air toxics emissions associated with individual cancer risk thresholds and population risks
Source: Environ Health. 2009 May 8;8:22. doi: 10.1186/1476-069X-8-22 (PMC2687437; doi:10.1186/1476-069X-8-22)
Supplement: Additional file 5 — Benzene intake fractions vs. population within 50 km of airports. This figure shows how benzene intake fraction increases approximately linearly with population within 50 km from airports. [file 1476-069X-8-22-S5.doc]

Figure S4 Benzene intake fractions vs. population within 50 km of airports

Note: the arrow marks the outlier mentioned in the text
